# New generation biofuel: continuous acidogenesis of sucrose-raffinose mixture simulating vinasse is promoted by γ-alumina pellets

**DOI:** 10.1186/s13068-015-0255-6

**Published:** 2015-05-07

**Authors:** Katerina Lappa, Panagiotis Kandylis, Nikolaos Bastas, Stavros Klaoudatos, Nikolaos Athanasopoulos, Argyro Bekatorou, Maria Kanellaki, Athanasios A Koutinas

**Affiliations:** Food Biotechnology Group, Department of Chemistry, University of Patras, 26500 Patra, Greece; B.G. Spiliopoulos S.A, 26500 Patra, Greece; I. Athanasopoulos and Co, 106b Lontou Str, 26224 Patra, Greece

**Keywords:** γ-Alumina, Acidogenic fermentation, Butyric acid, Lactic acid, Ethanol, Volatile fatty acids, Vinasse, Raffinose

## Abstract

**Background:**

This investigation comprises a contribution on the production of a new generation biofuel using the industrial liquid waste of bioethanol distilleries, known as vinasse. This study focuses on the exploitation of vinasse as an acidogenesis substrate for volatile fatty acids and simultaneous ethanol production. These products can be used for ester production, which is the new generation biofuel. Therefore, the aims of the present study are (i) to examine any promotional effect of γ-alumina on acidogenesis of a sucrose-raffinose mixture simulating vinasse, (ii) to study the operational stability of the continuous acidogenesis of sucrose and raffinose and subsequently of vinasse, and (iii) to determine the volatile fatty acid chemical composition and ethanol formation.

**Results:**

Batch acidogenesis of sucrose and raffinose mixtures showed that γ-alumina promoted fermentation leading to an increase in the volatile fatty acid yield factor from 40% to 95% compared to free cells. The application of the system in continuous mode for more than 3 months showed that the continuous volatile fatty acid productivity obtained was higher than 7 g/L/day. Lactic acid was the predominant acid when sucrose and raffinose were used while butyric acid in the case of vinasse. The highest volatile fatty acid concentration reached was 19 g/L for vinasse.

**Conclusions:**

A promoting effect of γ-alumina in acidogenic fermentation of sucrose-raffinose and vinasse is reported. Continuous acidogenesis of sucrose-raffinose mixtures and vinasse using γ-alumina with immobilized cells showed high operational stability (more than 3 months). These findings result in easy scale up of the process that will produce a high annual added value of $11,000,000 in a daily bioethanol production plant of 50,000 L.

## Background

In the European Union, about 29% of the total ethanol produced comes from industries using molasses [1]. After ethanol recovery, a liquid residue known as vinasse is produced (9 to 14 L per liter of ethanol) with a very high organic load [2,3]. The treatment and disposal of vinasse are very important since it may cause serious environmental problems due to its high chemical oxygen demand (COD).

Several methods have been proposed for the treatment of vinasse prior its disposal. Among them, fertirrigation (fertilization and irrigation) is the method with most applications [4], but several problems related to soil and groundwater contamination have been reported [5,6]. Other methods of vinasse disposal, but too expensive, are vinasse recirculation and vinasse concentration for volume reduction [7].

A biorefinery concept for vinasse exploitation includes its use as a substrate for fermentation and the production of value-added co-products, such as volatile fatty acids and ethanol, for application in novel biofuel production. This approach could reduce the problem of vinasse disposal and enhance the sustainability of sugarcane-to-ethanol plants. The use of volatile fatty acids and ethanol for the production of esters is very promising as a novel biofuel. In the frame of this possibility, it was reported that in the acidogenesis of glucose in a batch mode using anaerobic mixed culture, volatile fatty acids and ethanol were formed [8]. This gives the possibility for simultaneous production of the two chemicals that are necessary for the esterification reaction. The use of such esters in a homogeneous charge compression ignition engine gave promising results for use of such alternative liquid biofuels produced by anaerobic treatment of waste materials [9]. Furthermore, the porous γ-alumina was found to be an effective support for cell immobilization [10] and promoter of methane [11] and alcoholic [12] fermentations. Based on these properties of γ-alumina, it was recently found that this material promoted anaerobic acidogenic fermentation of glucose as a model compound both in batch and continuous modes, leading to accumulation of mainly volatile fatty acids (VFAs) and ethanol [13,14].

Therefore, the aims of the present study are (i) to examine any promotional effect of γ-alumina on the acidogenesis of a sucrose-raffinose mixture simulating vinasse, the liquid waste of alcohol distillery; (ii) to study the operational stability of the continuous acidogenesis of sucrose and raffinose and subsequently of vinasse; and (iii) to determine the VFA chemical composition and ethanol formation.

## Results and discussion

### Rationale of the investigation

The last decades, research efforts have been started, aiming to produce a new generation biofuel based on ester production from organic acids of the acidogenic fermentation using anaerobic mixed culture in the fermentation of glucose, resulting also in the simultaneous production of ethanol [8]. Furthermore, we discovered the promotional effect of γ-alumina in the acidogenesis of glucose [13] and under conditions favoring butyric acid production in the same fermentation of glucose medium [14]. These works open the way for the production of the new generation biofuel from wastes of the food production industry. Vinasse, the liquid waste of ethanol distilleries with high production capacity, contains sucrose and raffinose as residual sugar. Therefore, a study of acidogenesis of a model mixture of sucrose and raffinose is necessary to be carried out, as a precursor of vinasse treatment. This investigation’s objectives are to show any promotional effect by γ-alumina, as compared with free cells in the acidogenesis of sucrose and raffinose mixtures; to examine the chemical composition of VFA and ethanol formation in continuous processing; and to study the effectiveness of a γ-alumina-supported biocatalyst in the continuous acidogenesis of vinasse after adaptation of biocatalyst in a synthetic medium containing sucrose and raffinose found in sugarcane and sugar-beet vinasse. To achieve the objectives, batch and continuous acidogenic fermentations were performed and samples were analyzed for ethanol, acetic acid, propionic acid, butyric acid, lactic acid, succinic acid, malic acid, and valeric acid.

### Effect of γ-alumina on VFA and ethanol production using sucrose and raffinose mixtures

**Figure 1:**
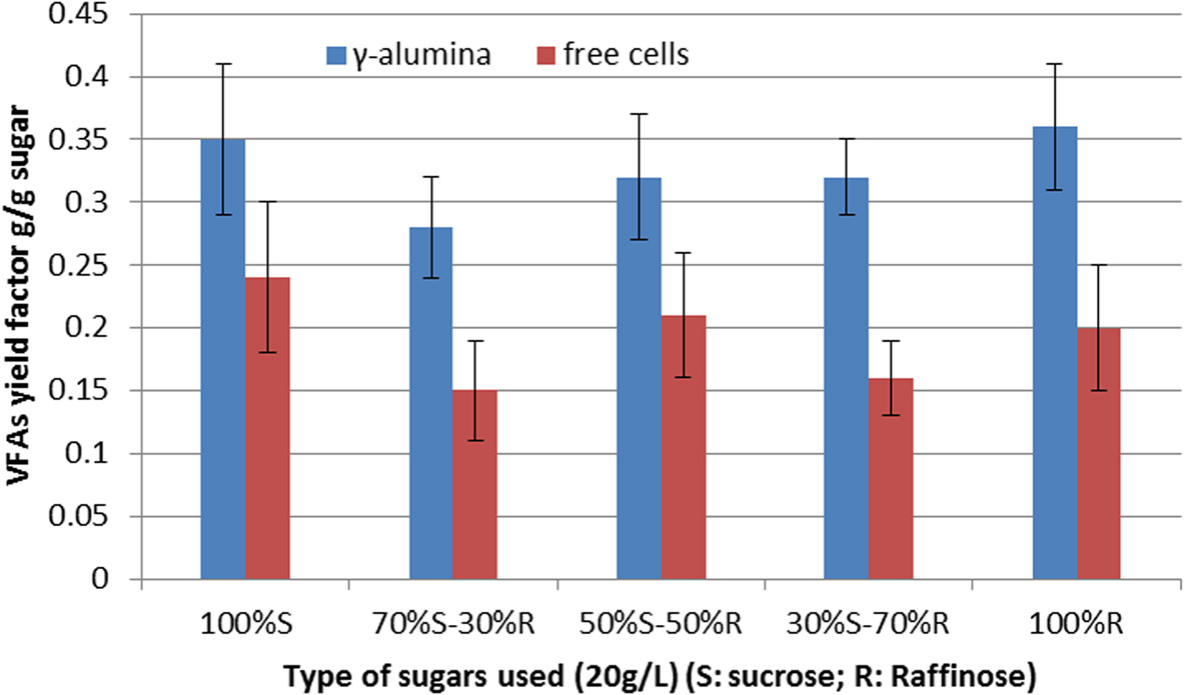
VFA yield factor during batch acidogenesis. Comparison of VFA yield factor during batch acidogenesis using γ-alumina-supported mixed anaerobic culture versus composition of sugars at 37°C and initial pH 7; suc: sucrose, raf: raffinose, vinasse diluted from concentrate, VFAs: volatile fatty acids.

Batch acidogenesis was performed at 37°C and a pH value of 7 as the optimum conditions were obtained. Mixtures of sucrose and raffinose (20 g/L) were used in order to simulate vinasse. The residual sugar in vinasse is less than 10 g/L containing sucrose and raffinose. However, the total organic material in vinasse is higher due to amino acid content, natural colorings, and so on. This is estimated to be a total organic mass of more than 20 g/L. Firstly, sucrose was used and then raffinose was added in various concentrations in order to obtain adaption to the sugars before vinasse treatment, which contains these carbohydrates. The simulation of vinasse was attempted with carbohydrates and yeast extract addition which is rich in amino acids. γ-Alumina increased the VFA yield factor in all mixtures of sucrose and raffinose from 40% to 95% as compared with free cells (Figure 1). The lower improvement was for sucrose, while the higher was for raffinose. Table 1 shows results of batch acidogenesis of sucrose and raffinose mixtures using immobilized cells on porous γ-alumina. VFAs formed in all repeated batch fermentations, proving the suitability of the biocatalyst for continuous mode operation. All sucrose and raffinose have been bioconversed in all batches and all mixtures. The yield factor varies from 0.20 to 0.42 g/g using an initial sugar concentration of 20 g/L. VFA concentration was from 4 to 8.49 g/L while ethanol formation did not exceed 1.4 mL/L. VFAs that were formed were acetic acid, propionic acid, butyric acid, lactic acid, and valeric acid. Lactic acid had a higher concentration in comparison with other acids. These results encouraged the study of continuous acidogenesis by immobilized cells on γ-alumina in order to achieve the aforementioned objectives.

**Table 1:** **Batch acidogenesis of sucrose and raffinose mixtures using γ-alumina-supported anaerobic mixed culture**

**Initial sugar composition**	**Repeated batch**	**Final pH**	**Ethanol (mL/L)**	**Acetic acid (g/L)**	**Propionic acid (g/L)**	**Butyric acid (g/L)**	**Lactic acid (g/L)**	**Succinic acid (g/L)**	**Malic acid (g/L)**	**Valeric acid (g/L)**	**Total VFAs (g/L)**	**Residual sugar (g/L)**	**Sugar conversion (%)**	**VFA yield (g/g)**
100% sucrose	1	5.6	0.55	1.21	0.29	1.31	1.23	-	-	-	4.04	0	100	0.20
	2	5.1	0.44	1.73	1.15	1.29	1.88	-	-	2.80	8.85	0	100	0.44
	3	4.7	0.34	1.50	0.79	1.05	3.43	-	-	1.43	8.20	0	100	0.41
70% sucrose 30% raffinose	1	5.6	0.74	1.44	0.69	2.56	0.63	-	-	-	5.32	0	100	0.27
	2	5.2	0.40	1.73	1.24	1.96	0.70	-	-	-	5.63	0	100	0.28
	3	4.9	0.20	0.99	0.64	0.94	0.81	-	-	2.33	5.71	0	100	0.29
50% sucrose 50% raffinose	1	5.4	1.04	1.19	-	1.36	1.45	-	-	-	4.00	0	100	0.20
	2	5.0	0.85	1.43	0.53	1.51	1.36	-	-	2.64	7.47	0	100	0.37
	3	4.7	0.30	1.04	0.47	1.01	3.77	0.86	0.94	-	8.09	0	100	0.40
30% sucrose 70% raffinose	1	5.5	0.98	1.53	0.35	1.77	1.09	0.86	-	-	5.60	0	100	0.28
	2	5.1	0.47	1.49	0.58	1.87	0.98	-	-	-	4.92	0	100	0.25
	3	4.8	0.29	1.39	0.68	1.55	2.36	-	-	2.32	8.30	0	100	0.42
100% raffinose	1	5.2	1.4	2.63	1.55	3.20	1.11	-	-	-	8.49	0	100	0.42
	2	4.9	0.6	1.11	0.51	1.34	1.36	-	-	2.83	7.15	0	100	0.36
	3	4.5	0.1	0.32	0.37	0.75	4.53	-	-	-	5.97	0	100	0.30

### VFA and ethanol production in continuous acidogenesis of sucrose and raffinose mixtures

**Figure 2:**
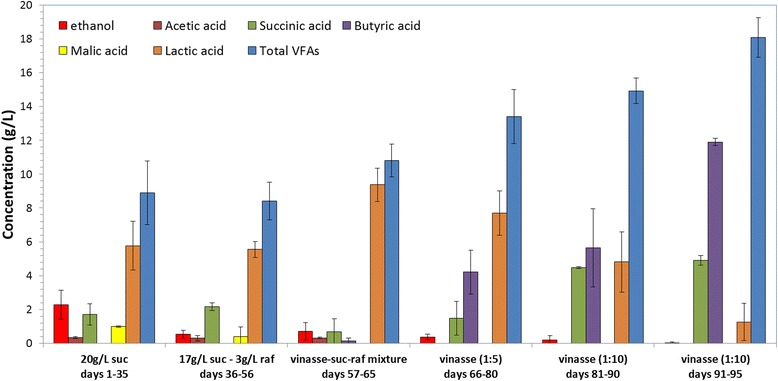
VFA and ethanol production during continuous acidogenesis. Ethanol and VFA composition of the effluent during the continuous acidogenesis using γ-alumina-supported mixed anaerobic culture versus fermentation time and different compositions of influent at 37°C and initial pH 7; suc: sucrose, raf: raffinose, vinasse diluted from concentrated, VFAs: volatile fatty acids.

**Figure 3:**
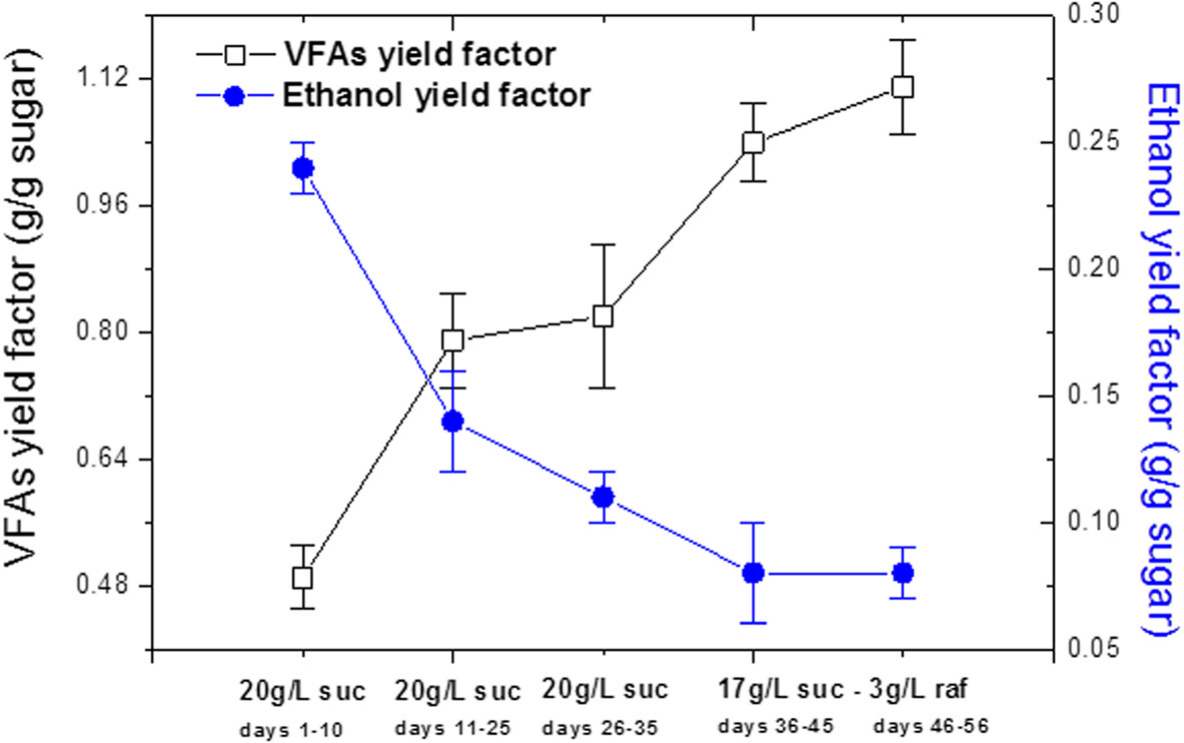
VFA and ethanol yield factor during continuous acidogenesis. Yield factors during the continuous acidogenesis using γ-alumina-supported mixed anaerobic culture versus fermentation time and different compositions of influent at 37°C and initial pH 7; suc: sucrose, raf: raffinose, VFAs: volatile fatty acids.

**Figure 4:**
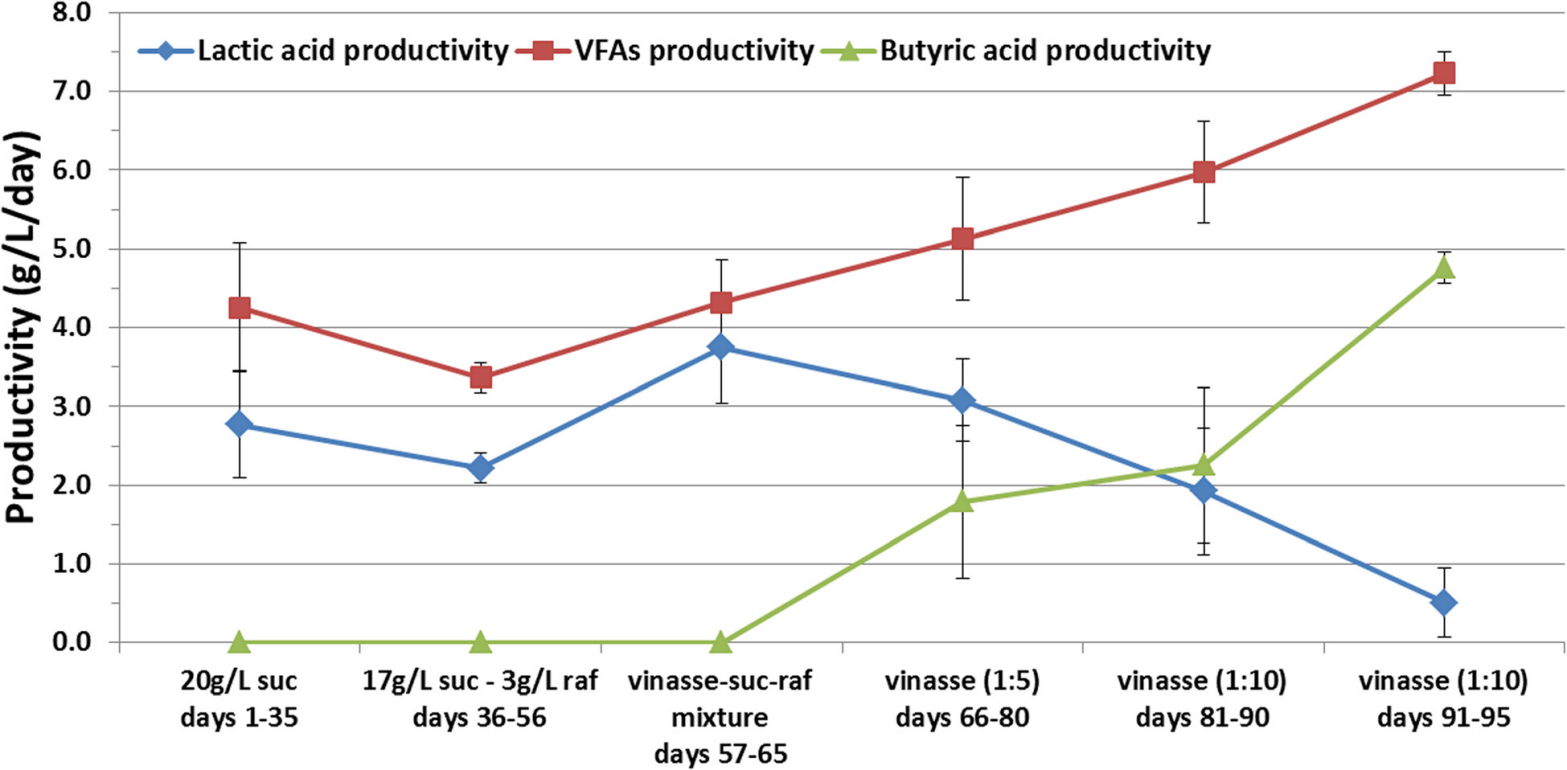
VFA, lactic acid, and butyric acid productivities versus time in continuous acidogenesis. Productivities during the continuous acidogenesis using γ-alumina-supported mixed anaerobic culture versus fermentation time and different compositions of influent at 37°C and initial pH 7; suc: sucrose, raf: raffinose, vinasse diluted from concentrate, VFAs: volatile fatty acids.

Continuous acidogenesis was performed using successively sucrose, sucrose-raffinose mixtures, and vinasse (Figure 2). Lactic acid was produced at the highest concentration as compared with acetic acid, butyric acid, succinic acid, and malic acid. Lactic acid, in continuous fermentation of sucrose and sucrose-raffinose mixtures, accounts for 65% of total VFAs while butyric acid was not produced at all. Figure 3 illustrates that the VFA yield factor increased as the time proceeds and as the acidogenesis proceeds from sucrose to sucrose-raffinose mixtures. The ethanol yield factor is reduced as the continuous acidogenesis proceeds. The operational stability of the continuous mode acidogenesis remains constant and increases for a long period of time as indicated by the stability of VFA productivity in Figure 4. Conversion varies from 34% up to 78.8% (Tables 2 and 3). The residual sugar that remains makes it possible for the effluent from the bioreactor to pass through γ-alumina-supported yeast cells to increase ethanol content which is necessary for the esterification reaction.

**Table 2:** **Continuous acidogenesis of synthetic media using γ-alumina-supported anaerobic mixed culture**

**Influent**				**Effluent**											
**Feed**	**Initial sugar (g/L)**	**Flow rate (L/day)**	**Operation time (days)**	**pH**	**Ethanol (ml/L)**	**Acetic acid (g/L)**	**Lactic acid (g/L)**	**Succinic acid (g/L)**	**Malic acid (g/L)**	**Butyric acid (g/L)**	**Total VFAs (g/L)**	**Residual sugar (g/L)**	**Sugar conversion (%)**	**VFA yield (g/g)**	**VFA productivity (g/L/day)**
Sucrose	20	0.2	1	4.9	7.7	0.18	3.83	1.32	1.01	-	6.34				2.54
	20	0.2	2	4.8	8.9	0.21	4.21	1.36	1.01	-	6.79	5.44	72.8	0.47	2.72
	20	0.4	3	4.7	10.5	0.23	4.41	1.34	1.08	-	7.06				5.65
	20	0.4	4	4.8	5.7	0.33	3.89	1.01	1.01	-	6.24				4.99
	20	0.4	6	4.8	2.9	0.39	3.35	0.84	1.01	-	5.59	7.06	64.7	0.43	4.47
	20	0.3	7	4.7	1.1	0.38	3.32	0.91	1.00	-	5.61				3.37
	20	0.3	8	4.7	1.0	0.35	3.83	0.99	0.99	-	6.16				3.70
	20	0.3	9	4.7	1.0	0.34	4.60	1.09	0.99	-	7.02	7.60	62.0	0.57	4.21
	20	0.25	10	4.7	1.94	0.32	5.35	1.17	0.97	-	7.81				3.91
	20	0.25	11	4.6	1.96	0.35	6.61	1.26	0.97	-	9.18				4.59
	20	0.25	12	4.5	1.85	0.32	6.91	1.29	0.95	-	9.48	4.24	78.8	0.60	4.74
	20	0.25	13	4.6	2.11	0.40	7.59	1.34	0.97	-	10.29				5.15
	20	0.25	14	4.6	1.85	0.38	7.37	1.34	0.96	-	10.05				5.03
	20	0.25	15	4.5	1.75	0.38	8.16	1.47	0.96	-	12.96	6.88	65.6	0.99	6.48
	20	0.25	16	4.7	2.18	0.34	6.58	1.30	0.96	-	9.19				4.60
	20	0.25	17	4.7	2.33	0.38	6.70	1.34	0.98	-	9.39				4.70
	20	0.25	18	4.7	2.4	0.38	7.02	1.40	0.77	-	9.57	6.99	65.0	0.74	4.79
	20	0.25	21	4.7	2.28	0.37	6.19	1.38	0.99	-	8.93				4.47
	20	0.2	23	4.5	4.38	0.37	9.04	2.30	0.97	-	12.67	5.06	74.7	0.85	5.07
	20	0.2	24	4.6	3.46	0.41	7.51	2.59	0.99	-	11.50				4.60
	20	0.2	25	4.6	3.43	0.42	6.10	2.61	1.01	-	10.15				4.06
	20	0.2	26	4.6	2.96	0.42	5.82	2.58	1.01	-	9.83	8.12	59.4	0.83	3.93
	20	0.2	27	4.6	6.04	0.43	5.99	2.54	1.02	-	9.98				3.99
	20	0.2	28	4.6	5.78	0.38	5.67	2.34	1.02	-	9.41				3.76
	20	0.2	29	4.6	5.26	0.36	5.61	2.24	1.02	-	9.23	7.74	61.3	0.75	3.69
	20	0.2	31	4.7	4.28	0.37	5.50	2.12	1.02	-	9.01				3.60
	20	0.2	32	4.7	1.89	0.33	5.62	2.29	1.03	-	9.28				3.71
	20	0.2	33	4.7	1.27	0.36	5.57	2.37	1.03	-	9.33	7.67	61.6	0.76	3.73
	20	0.2	34	4.7	1.40	0.25	5.22	2.53	1.05	-	9.05				3.62
	20	0.2	35	4.7	1.46	0.33	5.58	2.70	1.06	-	9.67	9.79	51.0	0.95	3.87
Sucrose and raffinose	17 g sucrose + 3 g raffinose	0.2	37	4.7	0.60	0.24	6.38	2.52	1.17	-	10.31	10.3	51.5	1.06	4.12
		0.2	38	4.7	1.07	-	5.57	2.24	1.06	-	8.87				3.55
		0.2	39	4.7	1.29	-	6.41	2.66	1.18	-	10.26				4.10
		0.2	40	4.7	1.09	0.18	6.36	2.62	1.18	-	10.15	11.9	40.5	1.25	4.06
		0.2	41	4.7	0.78	0.34	6.12	2.37	1.17	-	10.00				4.00
		0.2	43	4.7	0.84	0.34	5.52	2.22	1.08	-	9.16				3.66
		0.2	45	4.8	0.74	0.34	5.21	2.03	-	-	7.59	10.8	46.0	0.82	3.04
		0.2	46	4.7	0.78	0.33	5.32	2.07	-	-	7.72				3.09
		0.2	47	4.6	0.54	0.31	5.35	2.05	-	-	7.71				3.08
		0.2	48	4.7	0.83	0.25	5.39	2.05	-	-	7.69				3.08
		0.2	50	4.7	0.41	0.28	5.05	2.08	-	-	7.41				2.96
		0.2	51	4.7	0.83	0.33	5.13	2.13	-	-	7.59	13.2	34.0	1.12	3.04
		0.2	52	4.6	0.45	0.79	5.10	1.97	-	-	7.86				3.14
		0.2	53	4.7	0.48	0.35	5.34	1.97	-	-	7.66	13.1	34.5	1.11	3.06
		0.2	54	4.7	0.19	0.31	5.45	1.99	-	-	7.75				3.10
		0.2	55	4.7	0.22	0.38	5.48	2.04	-	-	7.90				3.16
		0.2	56	4.8	0.32	0.35	5.13	2.00	-	-	7.49				3.00

**Table 3:** **Continuous acidogenesis of vinasse using γ-alumina-supported anaerobic mixed culture**

**Influent**			**Effluent**							
**Feed**	**Flow rate (L/day)**	**Operation time (days)**	**pH**	**Ethanol (ml/L)**	**Acetic acid (g/L)**	**Lactic acid (g/L)**	**Succinic acid (g/L)**	**Butyric acid (g/L)**	**Total VFAs (g/L)**	**VFA productivity (g/L/day)**
25% vinasse (1:5)	0.2	58	5.2	0.35	0.36	6.33	1.80	-	8.49	3.40
	0.2	59	5.3	1.09	0.38	6.41	1.07	-	7.86	3.14
50% vinasse (1:5)	0.2	60	5.4	0.49	0.28	9.28	0.61	-	10.79	4.32
	0.2	61	5.4	1.96	0.31	13.09	-	-	14.03	5.61
75% vinasse (1:5)	0.2	64	5.5	0.70	0.31	11.72	-	0.78	12.81	5.12
100% vinasse (1:5)	0.2	66	5.3	0.56	-	10.45	-	2.36	12.81	5.12
	0.2	67	5.3	0.34	-	9.33	-	8.22	17.55	7.02
	0.2	68	5.2	0.22	-	8.60	-	7.53	15.79	6.32
	0.2	69	5.4	0.48	-	7.06	-	2.36	9.42	3.77
	0.2	71	5.4	0.28	-	6.73	1.56	7.78	16.07	6.43
	0.2	72	5.3	0.44	-	6.81	-	-	6.81	2.72
	0.2	73	5.5	0.36	-	7.37	-	-	7.37	2.95
	0.2	76	5.4	0.76	-	6.46	4.40	2.36	13.22	5.29
	0.2	78	5.3	0.84	-	7.38	4.49	2.69	14.56	5.82
	0.2	80	5.5	0.61	-	6.81	4.34	3.45	14.60	5.84
100% vinasse (1:10)	0.2	81	6.1	0.89	-	7.66	4.60	4.10	16.36	6.54
	0.2	82	5.8	0.40	-	6.98	4.47	3.50	14.95	5.98
	0.2	84	6.0	0.16	-	6.93	4.52	4.02	15.47	6.19
	0.2	85	6.2	-	-	5.27	4.43	4.66	14.36	5.74
	0.2	86	6.3	0.17	-	4.45	4.44	5.75	14.64	5.86
	0.2	88	6.3	0.24	-	2.37	4.47	7.56	14.40	5.76
	0.2	89	6.4	-	-	-	4.44	9.85	14.29	5.72
	0.2	90	6.2	0.13	-	-	4.60	12.14	16.74	6.70
	0.2	91	6.5	-	-	1.88	4.93	11.71	18.52	7.41
	0.2	94	6.4	-	-	1.91	5.17	11.86	18.94	7.58
	0.2	95	6.3	-	-	2.01	5.07	12.16	19.24	7.70

### VFA production in continuous acidogenesis of alcohol distillery’s liquid waste

After more than 2 months of operation, vinasse was used as influent in the continuous system and the results are summarized in Table 3. Vinasse was prepared from concentrate after dilution. Firstly, a dilution ratio of 1:5 was used simulating the content of fresh vinasse. The use of vinasse caused an increase in the total VFA content with a mean value of 14 g/L (varying from 7 to 17 g/L) and changed the VFA composition (Table 3 and Figure 2). More specifically, the concentration of lactic acid decreased to a mean value of 7 g/L (accounting for 50% of total VFA content), and subsequently, butyric acid and succinic acid concentrations increased to 4 g/L for each one (accounting for 25% of total VFAs each). The system continued its operation using 1:5 vinasse for 15 days, and then a more diluted vinasse was used. This time, the dilution ratio was 1:10 from the concentrated vinasse which is similar to the 1:2 dilution from fresh vinasse. This led to a further increase in total VFA concentration to a mean value of 16 g/L reaching at the last fermentations even up to 19 g/L. Furthermore, the lactic acid concentration continued to decrease reaching a value of 2 g/L, and the corresponding mean values for butyric and succinic acids were 7 and 4.5 g/L, respectively. The percentage of each acid in the total VFA content was 63% for butyric acid, 26% for succinic acid, and 10% for lactic acid. The results of VFA, lactic acid, and butyric acid productivities versus fermentation time are presented in Figure 4, showing the decrease of lactic acid and subsequently increase of butyric acid productivity. The VFA productivity continuously increases, and especially when vinasse is used, it reaches values of more than 7 g/L/day.

### Scientific and technological consideration

γ-Alumina is a porous material with numerous applications in catalysis. Furthermore, it has been used in several fermentation systems such as methane production and alcoholic fermentation and has been proven to be a very promising promoter. Recently, γ-alumina had promotional effect on acidogenic fermentations of glucose showing improvement of ethanol and VFA production [13,14]. The present investigation also proves the promotional effect of γ-alumina on the acidogenic fermentation of sucrose and raffinose sugars which are contained in sugarcane and sugar-beet vinasse. Sucrose-raffinose mixture was used as a model substrate, simulating vinasse, and γ-alumina is also used as an immobilization support for the mixed anaerobic culture leading to increased cell densities in the bioreactor facilitating the repeated batch and continuous processing. Repeated batch processing of sucrose-raffinose mixtures shows the suitability of biocatalyst for continuous processing. However, the batch process gave a lower VFA yield factor and VFA concentration as compared with the continuous process, which had an operational stability for a long time. The adaptation of biocatalyst in sucrose-raffinose mixtures accommodates a rapid start-up for vinasse acidogenic fermentation. Vinasse treatment leads to an important VFA yield factor, and in the mixtures of VFAs produced, lactic acid and butyric acid predominate. The last is of technological importance due to their esters with ethanol which have increased boiling and ignition points and viscosity, simulating better gasoline and therefore being a more suitable fuel to be added in biodiesel. Moreover, the results clearly showed that lactic acid is the main VFA in the acidogenesis of sucrose, sucrose-raffinose mixtures, and sucrose-raffinose-vinasse mixtures, while butyric acid is the main VFA when only vinasse was used. The formation of butyric acid at high concentrations in continuous acidogenesis of vinasse complies with the result of favoring butyric acid formation of a previous study [14]. Ethanol is necessary for the esterification of VFAs and can be produced after mixing of the effluent containing VFAs with a liquid waste containing glucose, sucrose, or lactose and supplying it through a second continuous bioreactor for ethanol formation in the same solution with VFAs. The second bioreactor can contain γ-alumina-supported yeast [10] or kefir yeast cells [15]. Based on the results of this investigation, one ethanol distillery with 50,000 L daily ethanol production can produce from its liquid waste vinasse a maximum of 15,000 L of esters daily as a new generation biofuel. Therefore, the annual added value is estimated to be $11,000,000. More specifically, 50,000 L of ethanol will produce 575,000 L of vinasse, which will result, after acidogenesis, to 11,500 kg of acids daily. After esterification, these acids will result to 15,000 L of esters per day and therefore 5,475,000 L annually. If this replaces gasoline, it means an added value of almost $11,000,000. This added value will reduce bioethanol production cost by about 30%.

## Conclusions

The acidogenic fermentation of sucrose and raffinose is promoted by γ-alumina. Batch acidogenesis of sucrose-raffinose mixtures gave a lower concentration and yield factor of VFAs than the continuous process. Continuous acidogenesis of sucrose-raffinose mixtures using γ-alumina with immobilized cells has high operational stability, and the adaptation of a biocatalyst results to a rapid scale up for the acidogenesis of vinasse. Lactic acid is the predominant VFA when synthetic medium is used, and butyric acid predominates in VFAs produced when vinasse is used. The added value produced from the new generation biofuel as a bioproduct of an ethanol distillery will reduce its production cost by about 30%.

## Materials and methods

### Culture and growth media

Mixed bacterial anaerobic culture was obtained from an upflow anaerobic sludge blanket (UASB) bioreactor and was inoculated in a medium containing 50 g/L glucose, aqueous NH_3_ and 50% H_3_PO_4_ (to achieve a COD:N:P ratio of 100:5:1), NaHCO_3_ 4 g/L, and yeast extract 4 g/L, without pH adjustment [11]. Cell growth was carried out at 37°C. The medium was sterilized by autoclaving at 120°C for 10 min.

### Batch acidogenic fermentation of sucrose and raffinose mixtures

For the repeated batch fermentations, conical flasks were used containing immobilized mixed anaerobic culture on 100 g cylindrical γ-alumina and 100 mL of synthetic medium containing 20 g/L sugars. Fermentations were carried out at 37°C with an initial pH of 7. Synthetic medium with five different compositions of sugars was used containing 20 g/L sucrose, 14 g/L sucrose and 6 g/L raffinose, 10 g/L sucrose and 10 g/L raffinose, 6 g/L sucrose and 14 g/L raffinose, and 20 g/L raffinose. Each medium also contained aqueous NH_3_ and 50% H_3_PO_4_ (to achieve a COD:N:P ratio of 100:5:1), NaHCO_3_ 4 g/L, and yeast extract 4 g/L, with pH adjustment at 7 [11]. The media were sterilized by autoclaving at 120°C for 10 min.

### Cell immobilization and continuous anaerobic acidogenic fermentation of sucrose

The mixed culture was fixed on porous cylindrical γ-alumina pellets (1.40 m^2^/g surface area) to increase rate of fermentation and enable continuous processing. The experimental apparatus used for cell immobilization and for continuous acidogenic fermentation consisted of a 1.25-L glass tower reactor. The bioreactor was filled with 700 g of γ-alumina pellets and equal volumes of 20 g/L sucrose medium, and anaerobic culture was added. The bioreactor was placed in an incubator set at 37°C, and it was left to ferment for 2 days without feeding in order to achieve cell immobilization. Subsequently, fresh sucrose medium was pumped continuously into the bioreactor using a high-accuracy peristaltic pump, and a steady state was obtained in a few days. The bioreactor was pumped continuously and successively with sucrose, sucrose and raffinose mixture, and vinasse as described previously. Samples were taken at various time intervals and analyzed for volatile fatty acids, ethanol, and residual sugar by GC and HPLC as described below.

### Media for continuous acidogenic fermentation

During the continuous anaerobic fermentation, media of various sugar substrates were used successively. At the beginning, 20 g/L sucrose was used for 35 days, then a mixed medium of 17 g/L sucrose and 3 g/L raffinose was tested for 21 days and then concentrated vinasse (sugar-beet) after dilution with water (1:5 dilution for 15 days and 1:10 dilution for 15 days) was the last medium used. Each medium also contained aqueous NH_3_ and 50% H_3_PO_4_ (to achieve a COD:N:P ratio of 100:5:1), NaHCO_3_ 4 g/L, and yeast extract 4 g/L, with pH adjustment at 7 [11]. The media were sterilized by autoclaving at 120°C for 10 min.

### GC analysis for ethanol determination

Ethanol was determined on a Shimadzu GC-8A system (Shimadzu Corporation, Kyoto, Japan), with a Teknokroma HAYE SEP Q 80/100 column, a C-R6A Chromatopac integrator, a He as carrier gas (40 mL/min), and a FID detector. The injection port and detector temperature was 210°C. The column temperature was 130°C. Samples of 2 μL were injected directly into the column. Determinations were done by means of standard curves.

### HPLC analysis for VFA and residual sugar determination

Residual sugar was determined by high-performance liquid chromatography, using a Shimadzu chromatograph with a Nucleogel Ion 300 OA column, a LC-9A pump, a CTO-10A oven at 30°C, and a RID-6A refractive index detector. H_2_SO_4_ 0.008 Ν was used as mobile phase with a flow rate of 0.8 mL/min, and propanol-1 was used as an internal standard. A volume of 0.25 mL of the sample and 0.625 mL of a 1% (*v*/*v*) solution of propanol-1 were diluted to 25 mL. Then, 60 μL of the final solution was injected directly to the column. Residual sugar and concentrations were calculated using standard curves.

Volatile fatty acids were determined by high-performance liquid chromatography, using a Jasco chromatograph LC-2000 Plus (Jasco Analytical Instruments, Tokyo, Japan) with a Bio-rad Aminex HPX-87H column (7.8 mm ID × 300 mm, 9 μm particle size), a PU-2089 plus quaternary gradient pump, a Jasco CO-2060 Plus oven at 50°C, a MD-2018 plus photodiode array detector operated at 210 nm, and an autosampler AS 2050 plus. H_2_SO_4_ 0.008 Ν was used as mobile phase with a flow rate of 0.6 mL/min. The samples were filtered by a membrane filter of 0.45 nm, and all the data were processed with the ChromNav program.

## Abbreviations

COD: chemical oxygen demand
Raf: raffinose
Suc: sucrose
UASB: upflow anaerobic sludge blanket
VFAs: volatile fatty acids
